# Pathogen Infection and Host-Resistance Interactively Affect Root-Associated Fungal Communities in Watermelon

**DOI:** 10.3389/fmicb.2020.605622

**Published:** 2020-12-17

**Authors:** Lihui Xu, Mogens Nicolaisen, John Larsen, Rong Zeng, Shigang Gao, Fuming Dai

**Affiliations:** ^1^Institute of Eco-Environmental Protection, Shanghai Academy of Agricultural Sciences, Shanghai, China; ^2^Shanghai Key Laboratory of Protected Horticultural Technology, Shanghai, China; ^3^Department of Agroecology, Faculty of Technical Sciences, Aarhus University, Slagelse, Denmark; ^4^Instituto de Investigaciones en Ecosistemas y Sustentabilidad, Universidad Nacional Autónoma de México, Morelia, México

**Keywords:** Fusarium wilt of watermelon, mycobiome, root and rhizosphere, Illumina MiSeq, cultivar, disease resistance

## Abstract

Interactions of pathogen infection, host plant resistance, and fungal communities are poorly understood. Although the use of resistant watermelon cultivars is an effective control measure of watermelon wilt disease, fungal communities may also have significant effects on the development of the soil-borne pathogen complexes. We characterized the root and rhizosphere fungal communities associated with healthy and diseased watermelons of three different cultivars with different susceptibilities toward wilt disease by paired-end Illumina MiSeq sequencing. Thirty watermelon plants including highly wilt-resistant, moderately resistant, and susceptible cultivars were collected from a greenhouse, half of which showing clear wilt symptoms and the other half with no symptoms. Patterns of watermelon wilt disease and the response of the fungal communities varied among the three cultivars. The amount of the pathogen *Fusarium oxysporum* f. sp. *niveum* was higher in diseased root and rhizosphere samples, particularly in the susceptible cultivar, and was significantly positively correlated with the disease index of Fusarium wilt. Plant health had significant effects on root-associated fungal communities, whereas only the highly resistant cultivar had significant effects only on the rhizosphere fungal communities. Co-occurrence networks revealed a higher complexity of fungal communities in the symptom-free roots compared to diseased roots. In addition, networks from roots of the highly resistant plants showing symptoms had a higher complexity compared to the susceptible cultivars. Keystone species were identified for the plants with different symptom severity and the different cultivars in the root and rhizosphere, such as *Fusarium oxysporum, Monosporascus cannonballus*, and *Mortierella alpina*. Overall, the most important factor determining fungal communities in the roots was plant symptom severity, whereas in the rhizosphere, plant genotype was the most important factor determining fungal communities.

## Introduction

Fungi are an immensely diverse group of organisms that play crucial roles in plant growth as degraders of organic material to nutrients, as plant pathogens, or as beneficial organisms that suppress pathogens (Raaijmakers et al., 2009). Plant roots harbor a large diversity of microorganisms with an essential role in ecosystem functioning (Vandenkoornhuyse et al., 2007), and the plant microbiome thus represents one of the key determinants of plant health and productivity (Mendes et al., 2011; Berendsen et al., 2012; Turner et al., 2013; Berg et al., 2014; Beckers et al., 2017). The rhizosphere microbiome have beneficial functions for the host plant, including nutrient acquisition, stress tolerance and protection against soil-borne pathogens (Mendes et al., 2011; Pérez-Jaramillo et al., 2016).

Plant health is the result of complex interactions involving the plant, the physical and chemical soil environment, together with the associated microbiome including pathogens and non-pathogenic microorganisms. Microbial diversity in soil is one of the key drivers determining soil quality and soil suppressiveness (Garbeva et al., 2004). The structure of microbial communities associated with plant roots is affected by various key factors, such as plant species and cultivar, chemical and physical properties of the soil, and also by the general plant and soil health (Marschner et al., 2001; Berg and Smalla, 2009; İnceoğlu et al., 2012; Xu et al., 2012a; Mendes et al., 2018). Hence, interactions between fungal pathogens, host plants and the rhizosphere microbiome are key elements that are shaping a plant-protective microbiome or, under favorable conditions for the pathogens, leading to development of disease (Chapelle et al., 2016).

Fusarium wilt of watermelon [*Citrullus lanatus* var. *lanatus* (Thunb.) Matsum. & Nakai], caused by the fungal pathogen *Fusarium oxysporum* f. sp. *niveum* (FON), is a serious soil-borne disease threatening watermelon production around the world (Martyn, 2014). The pathogen induces symptoms such as vascular discoloration, especially around the crown and upper taproot, and withering and wilting of leaves followed by death of the vine or the whole plant. Watermelon wilting may, however, also be caused by *Monosporascus* (Cohen et al., 2000) which causes rapid wilting at the time of fruit maturity and which may result in total loss of the crop (Castro et al., 2020).

The use of resistant watermelon cultivars is an environmentally friendly and efficient control measure for watermelon wilt disease (Keinath et al., 2019). Elucidating the relationship between resistant cultivars and their associated microbial communities may support cropping systems and guide biological control strategies. The evaluation of plant lines for their interaction with rhizosphere microorganisms is also essential for future breeding programs (Bakker et al., 2012).

We characterized the fungal communities (the mycobiome) associated with watermelon cultivars having different levels of resistance against FON and having different levels of wilting symptoms. The objectives of the study were (i) to profile and compare fungal communities in plant roots and their rhizosphere, (ii) to compare fungal communities from watermelon cultivars having different resistance levels toward FON, and having different levels of wilting symptoms, and (iii) to identify fungal taxa that were responding to different FON infection levels. In order to understand dynamics in the fungal communities, we used co-occurrence network analysis which has previously been applied in microbial ecology to explore patterns of microbiome assembly (Barberán et al., 2012), and to identify keystone taxa (Berry and Widder, 2014).

## Materials and Methods

### Plant and Soil Sampling

Plant and rhizosphere samples were collected from three different watermelon cultivars: Shenkang 988 (highly resistant to FON), Lvfeng 99 (moderately resistant to FON), and Tianshan 8424 (susceptible to FON) grown in a plastic greenhouse located in Shanghai, China (31°07′36 ″N, 121°43′41″E). The field soil, in which watermelon had been grown the year before, was sandy and loam with a pH of 6.87, an organic matter content of 1.15%. The experiment covered an area of 300 m^2^ divided into 12 plots of 25 m^2^. Plantlets were transplanted into the greenhouse after true leaves had emerged. Every cultivar was planted in four plots and each plot was planted with 25 seedlings. The experiment was organized in a completely randomized block design. Disease severity of all plants was scored based on the rate of shoot wilting for each individual plant on a 5-grade scale as follows: 0 = healthy, no visible symptoms; 1 = 1–10% of symptomatic leaves (leaves with mild chlorosis and wilting); 2 = 11–25% of symptomatic leaves (leaves with moderate chlorosis and wilting); 3 = 26–50% of symptomatic leaves (leaves with severe chlorosis and wilting); and 4 = plant completely wilted (Ren et al., 2015). Disease incidence was defined as the percentage of diseased leaves and stems (Matsumoto, 2012) and calculated as the percentage of diseased plants over the total number of watermelon plants (Wu et al., 2009). Disease index of each treatment was calculated according to the formula as shown in Świąder et al. (2002): Disease Index = Σ(nv)100/NV, where: n = degree of infection rate according to 5-grade scale, v = number of plants in a category, V = total number of plants screened, N = highest degree of infection rate.

Based on visual inspection and rating of each plant, five plants with similar disease severity symptoms and five plants with no visible symptoms were harvested from each cultivar at the maturation stage. Each individual plant was collected with its rhizosphere soil. Rhizosphere soil was then collected from the plants by carefully brushing roots and removing root hairs from the collected soil. Finally, the remaining soil was removed from the roots by gentle washing in sterile water. The resulting 30 root samples and 30 rhizosphere soil samples were kept at −80°C until processing.

### DNA Extraction and Quantitative PCR Analysis

Freeze-dried root and rhizosphere soil samples were homogenized in a Geno/Grinder 2010 (SPEX SamplePrep, Metuchen, NJ, USA) for 6 × 1 min in plastic tubes with two steel balls prechilled in liquid nitrogen between the six subsequent homogenizations. DNA was extracted from 100 mg of root material using the DNeasy Plant Mini Kit (Qiagen GmbH, Hilden, Germany) and from 250 mg of rhizosphere soil using the DNeasy PowerSoil Kit (Qiagen GmbH, Hilden, Germany) according to the manufacturer's instructions. The DNA concentrations of all samples were measured by NanoDrop 2000 spectrophotometer (Thermo Fisher Scientific, Inc., Waltham, MA, USA), and adjusted to 20 ng/μL for the normalization of subsequent procedures.

Quantitative PCR analysis (qPCR) was performed to quantify the number of gene copies of FON in roots and rhizosphere samples of the three watermelon cultivars. The amplification reactions were run using the primers fp7315 (5′-CGC TCG CTA TAA TTC AAA CG-3′) and fp7332 (5′-GGA GGA GCA CTA CAA CTA AT-3′), which targets Fom effector 7 gene generating an amplicon size of 139 bp (van Dam et al., 2018). In a final volume of 20 μL, reactions contained 10 μL of PowerUp™ SYBR™ Green Master Mix (Applied Biosystems by Thermo Fisher Scientific, Inc., Foster City, CA, USA), 1.25 μM of each primer, and 1 μL of DNA template in a MicroAmp™ Optical 96-Well Reaction Plate. DNA amplification was performed with an initial denaturation temperature at 94°C for 2 min, followed by 40 cycles of 94°C for 30 s, 54°C for 30 s, and 72°C for 40 s with final melting curve of 95°C for 15 s, 60°C for 1 min and temperature increasing to 95°C for 15 s, with data collecting at 0.05°C/s. Reactions were carried out on the QuantStudio™ 6 Flex System (Applied Biosystems by Thermo Fisher Scientific, Inc., Foster City, CA, USA). The Ct values (cycle threshold) were used as standards for determining the amount of DNA template in each sample. Standard curves were produced using a cloned fragment of the corresponding gene from FON. Gene fragments were quantified in the Tecan Infinite M200 PRO NanoQuant (Tecan Austria GmbH, Grödig, Austria) and diluted (10^−2^ to 10^−6^ genes/μL) to generate the standard curve. Samples together with positive and negative controls were run in triplicate.

### Library preparation for Illumina MiSeq Sequencing

The ITS1 region of the fungal internal transcribed spacer (ITS) was amplified using the ITS1F (5′-CTT GGT CAT TTA GAG GAA GTAA-3′) and ITS2 (5′-GCT GCG TTC TTC ATC GAT GC-3′) primers (Mueller et al., 2014). The primers used for final sequencing consisted of an appropriate Illumina adapter, pad linker, gene-specific primer and a 6-nt unique barcode attached to the forward and reverse primer.

PCR reactions contained 1 × PCR reaction buffer, 0.25 mM dNTPs, 0.2 μM each primer, 1U of TransStart Fastpfu DNA polymerase (TransGen AP221-02), and 10 ng of DNA template with a final volume of 20 μL. All amplifications were performed in a GeneAmp PCR System 9700 thermal cycler (Applied Biosystems by Thermo Fisher Scientific, Inc., Foster City, CA, USA) using an initial DNA denaturation step of 95°C for 3 min, followed by 32 cycles of denaturation at 95°C for 30 s, annealing at 55°C for 30 s, extension at 72°C for 45 s, and a final elongation at 72°C for 10 min. PCR products were analyzed by gel electrophoresis in a 2% agarose gel. Each sample was amplified in triplicate, pooled and purified using AxyPrep DNA Gel Extraction Kit (Axygen, Tewksbury, MA, USA). PCR products were normalized after quantifying them with a QuantiFluor™-ST Handheld Fluorometer with UV/Blue Channels (Promega Corporation, Madison, WI, USA). Paired-end sequencing (2 × 250 bp) was conducted on an Illumina MiSeq sequencer at Shanghai Majorbio Bio-pharm Technology Co., Ltd. (Shanghai, China).

### Sequence Processing

Raw sequences were assembled and processed using QIIME 1.8 (Caporaso et al., 2010). The raw paired-end sequences were merged using the FLASH V1.2.7 software (Magoč and Salzberg, 2011) with a minimum overlap of 10 bp. PCR artifacts were denoised by eliminating low-quality sequences using Trimmomatic (Bolger et al., 2014). The chimeric sequences were removed by UCHIME (Edgar et al., 2011). The quality checked sequences were clustered into operational taxonomic unites (OTUs) following the UPARSE pipeline through USEARCH at the 97% similarity threshold for generating OTUs (Edgar, 2013). The most abundant sequence in each OTU was chosen as a representative sequence and were classified against the UNITE Fungal database (Abarenkov et al., 2010) and the non-redundant Genbank database (Benson et al., 2011; Kõljalg et al., 2013). OTUs at 97% sequence similarity that could not be identified using the UNITE Fungal database were recovered by BLAST searching against the GenBank database. OTUs defined at 97% sequence similarity were used to generate rarefaction curves and to estimate the richness of communities (Chao1) (Chao et al., 2005), diversity (Shannon), and Good's coverage by Mothur (version v.1.30.1) (Schloss et al., 2009). Either normalized sequence counts from individual subsamples or the average counts of five subsamples were used for further analyses. All raw sequence data were deposited in the Sequence Read Archive (SRA) database at NCBI under the BioProject number PRJNA643411.

### Statistical Analyses

The levels of significance (*P* < 0.05) of disease incidence and disease severity was examined using Kruskal-Wallis test. Two-way analysis of variance (ANOVA) was performed to analyze the levels of significance of the individual factors and their interactions in terms of effects on the number of FON gene copies, OTU richness and diversity, and most abundant OTUs from roots and rhizosphere. *P*-values were corrected for multiple testing using Bonferroni corrections. Post-ANOVA mean comparisons were performed with least significant difference (LSD) values. Pearson correlation analysis was conducted to examine the relationship between the number of FON gene copies by qPCR and the number of *F. oxysporum* reads by sequencing.

To examine dissimilarities in community composition, β-diversity distance matrices of OTUs in roots and rhizosphere were obtained using Bray-Curtis dissimilarity and visualized using principal coordinates analysis (PCoA) conducted by the vegan package of R, respectively. Analysis of similarities (ANOSIM) (Clarke, 1993) and permutational multivariate analysis of variance (PERMANOVA) with a permutation number of 999 in the vegan package was performed using relative abundance data of individual OTUs, in order to determine the effects of cultivar and symptom severity on fungal communities.

Indicator species analysis (ISA) was used to identify indicator species associated with two categories (symptom severity and cultivar) using the “indicspecies” package of R (Dufrêne and Legendre, 1997). The association index IndVal was used and inspected the two indicator value components, including Component “A” (the specificity or positive predictive value of the species as indicator) and Component “B” (the fidelity or sensitivity of the species as indicator) (Cáceres and Legendre, 2009). Indicator OTUs with a *P* < 0.05 as well as fidelity and specificity values ≥0.8 were considered valid, ensuring that potential indicator OTUs are both widely distributed and restricted to every category (Dufrêne and Legendre, 1997; Salazar et al., 2015).

Network analyses were performed to assess the complexity of the interactions within microbial community. Pearson correlation coefficients were calculated based on the 100 most abundant OTUs, accounting for 99% of the total relative abundance. Statistically significant (*P* < 0.05) correlations with a magnitude of > 0.7 or < − 0.7 were included into the network analyses. The nodes in the reconstructed network represent taxa at OTU level, whereas the edges represent significant positive or negative correlations between nodes. The network graphs were made based on number of nodes, number of edges, modularity, number of communities, average node connectivity, average path length, diameter, and cumulative degree distribution. Network visualization and property measurements were conducted with the interactive platform Gephi (Bastian et al., 2009).

All the analyses were performed using R 3.6.1 (R Development Core Team, 2011) and SPSS 26.0 (IBM Corp., Armonk, NY, USA). Graphs were rendered in R and Sigmaplot 14.0 (Systat Software, Inc., San Jose, CA, USA).

## Results

### Watermelon With Symptoms, and Susceptible Cultivars, Had Higher FON Infection Levels

**Figure 1:**
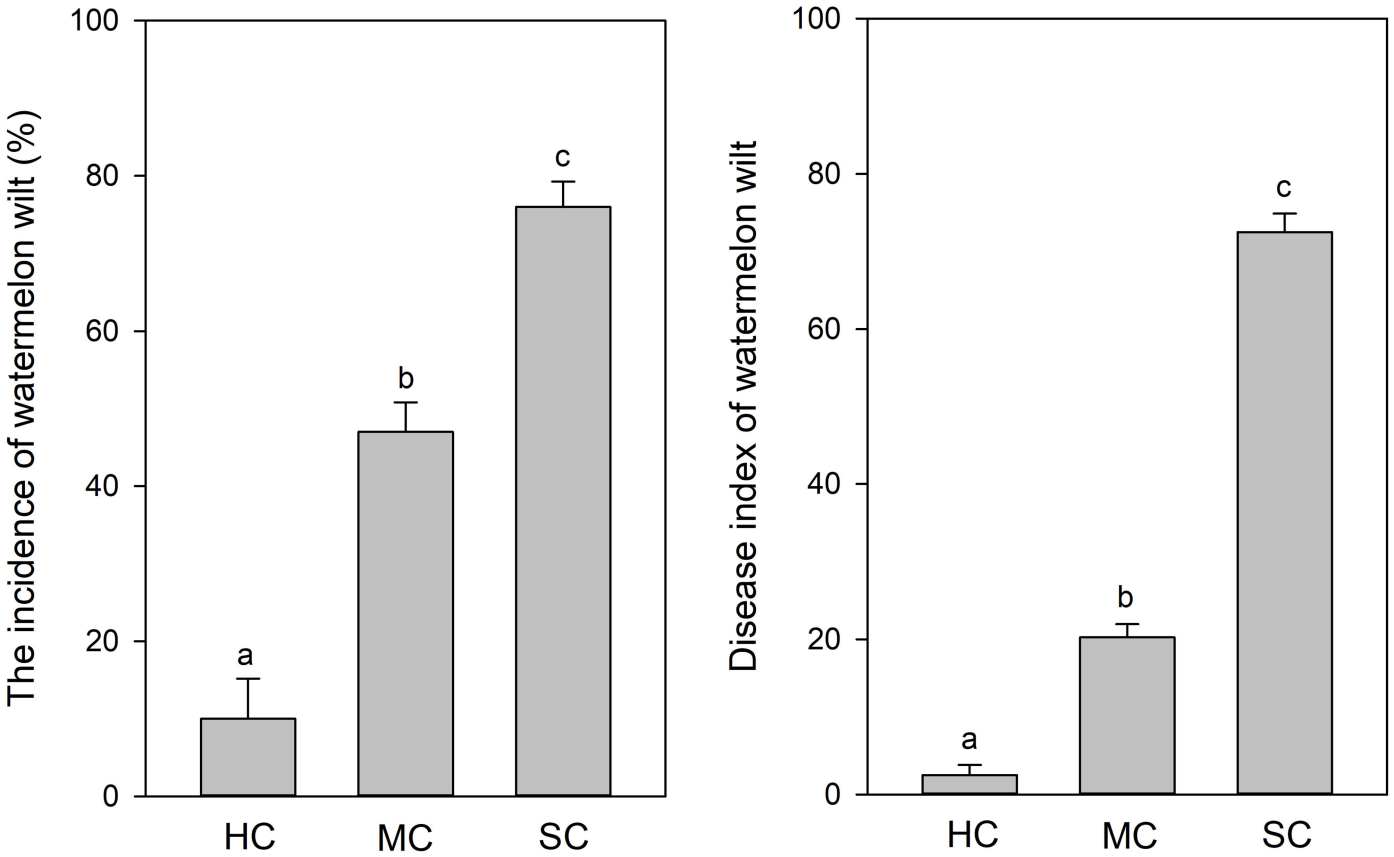
Incidence and severity of watermelon wilt in the greenhouse with four replicate experimental plots of every cultivar. Each experimental plot had 25 watermelon plants. HC, highly resistant cultivar; MC, moderately resistant cultivar; SC, susceptible cultivar. Different letters indicate significant difference among different cultivars (*n* = 100, *P* < 0.05).

According to visual observations, diseased plants occurred in large patches among individuals of the susceptible cultivar “Tianshan 8424.” In contrast, only weak symptoms of wilt and curled leaves were observed in the plots with the moderately resistant cultivar “Lvfeng 99” and the highly resistant cultivar “Shenkang 988.” Significant differences of disease incidence were observed among the different cultivars (*P* = 0.0). The highly resistant cultivar “Shenkang 988” showed significantly lower disease incidence (10%), compared to the moderately resistant “Lvfeng 99” (47%) and the susceptible “Tianshan 8424” (76%). Disease indices were significantly lower in the highly resistant cultivar (2.5), compared to the moderately resistant cultivar (20.3), and the susceptible cultivar (72.5) (*P* = 0.0) (Figure 1).

**Figure 2:**
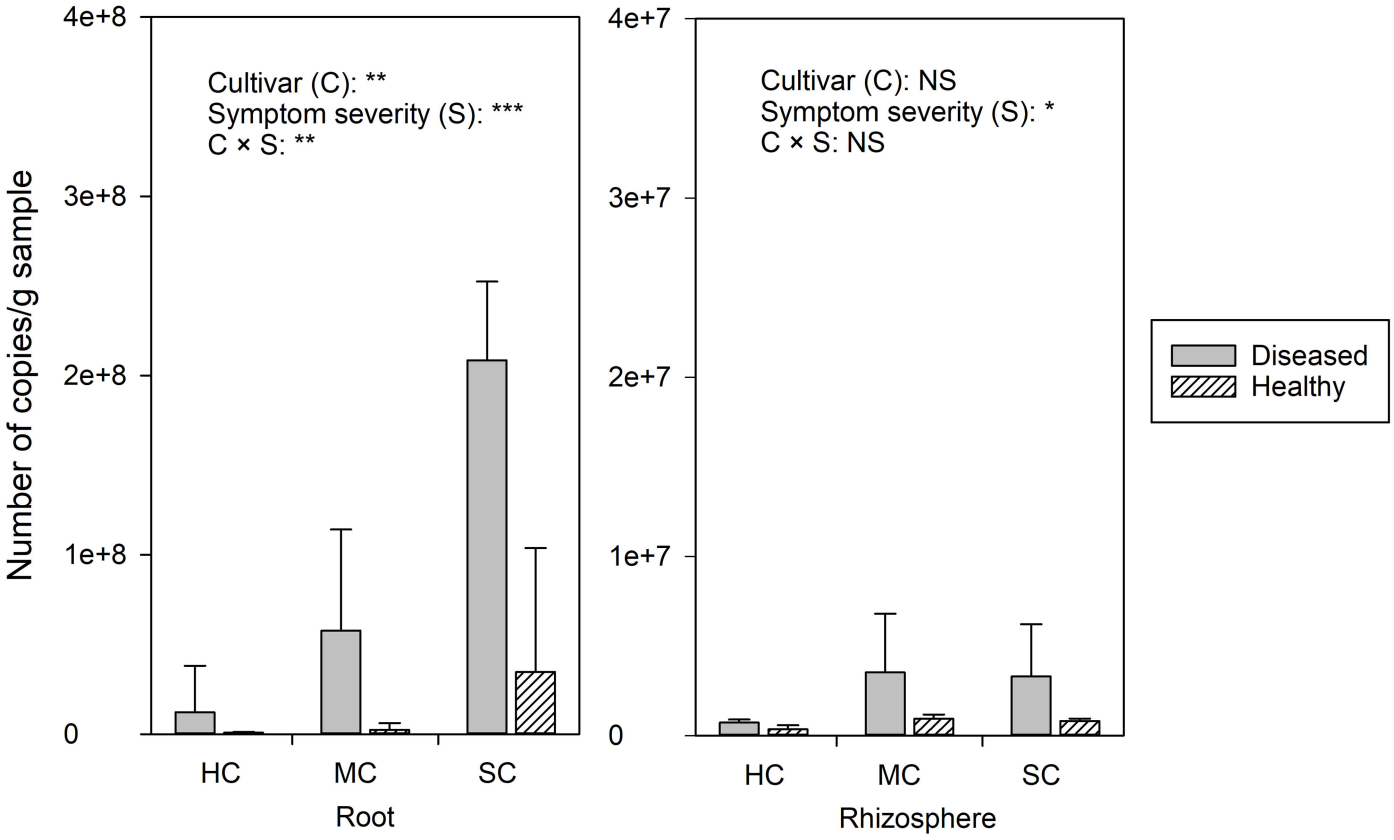
The abundance of *Fusarium. oxysporum* f. sp. *niveum* detected by qPCR from watermelon roots and rhizosphere sampled from diseased (D) and healthy (H) watermelon of different cultivars (HC, highly resistant cultivar; MC, moderately resistant cultivar; SC, susceptible cultivar). Significant differences between treatment means according to multiple range test after Bonferroni correction are listed (*n* = 5). *P < 0.05; **P ≤ 0.01; ***P ≤ 0.001; and NS, not statistically significant.

FON quantities detected by qPCR was notably higher in roots compared to the rhizosphere (Figure 2). Significant differences of FON gene copy numbers between cultivars (with the highest numbers in the susceptible cultivar) (*P* = 0.006) and symptom severity (with the highest numbers in severely affected plants) (*P* = 0.0) were observed in the roots. In the rhizosphere, a moderately increased amount of FON gene copy numbers was observed in the plants with symptoms (*P* = 0.011) (Figure 2). Pearson correlation analysis showed a clear relationship between the number of FON gene copies and the number of *F. oxysporum* reads in the roots (*r* = 0.871, *P* = 0.024) and rhizosphere (*r* = 0.657, *P* = 0.035).

### Species Richness of Fungal Communities Is Dramatically Affected by Symptom Severity and by Cultivar Resistance

**Figure 3:**
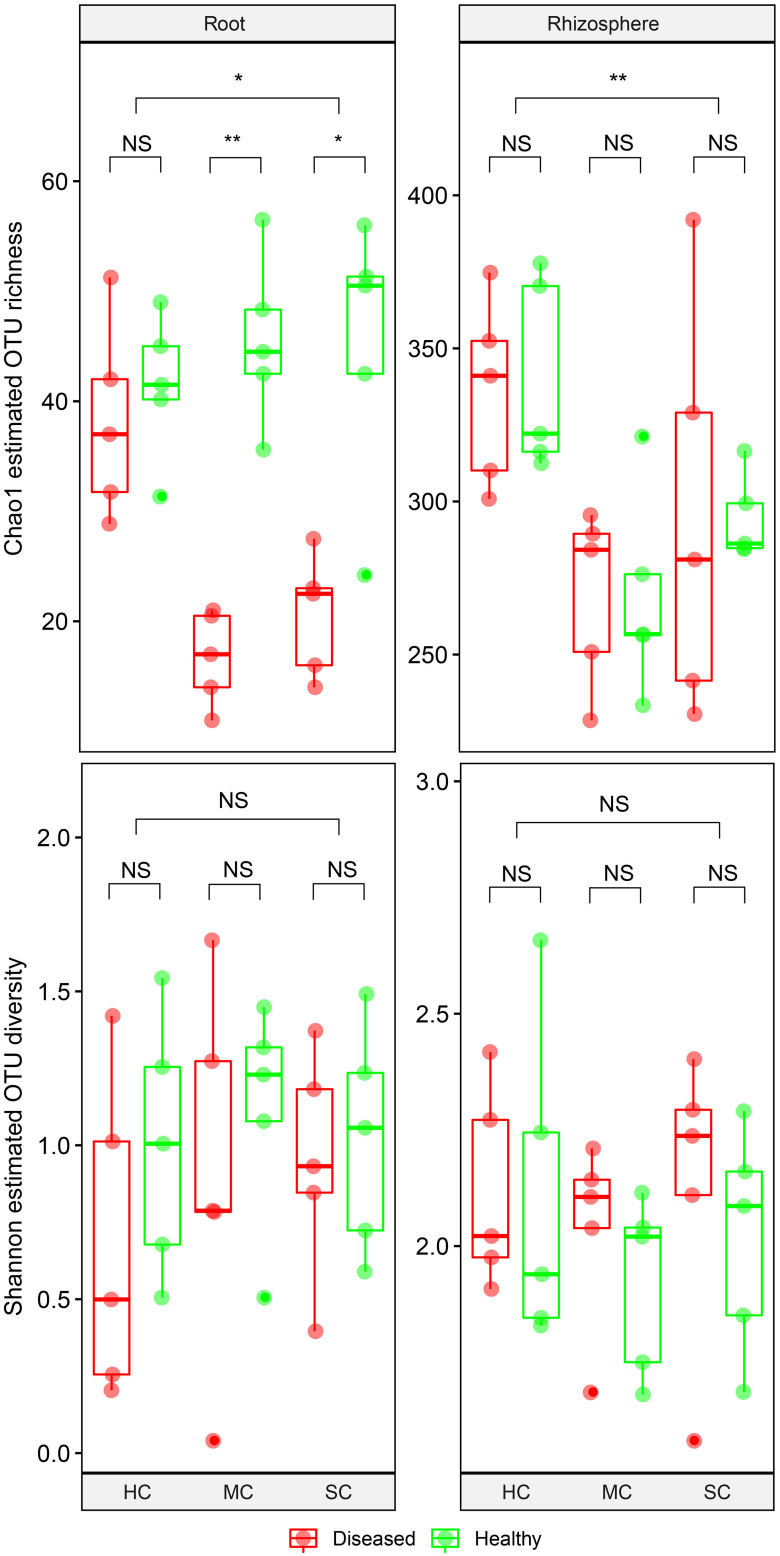
Boxplot showing alpha diversity estimation of the fungal communities including the Chao1 richness estimator and Shannon diversity index. The points represent samples from diseased (D) and healthy (H) watermelon of different cultivars (HC, highly resistant cultivar; MC, moderately resistant cultivar; SC, susceptible cultivar) from root and rhizosphere. Upper, middle, and lower lines represent first quartiles, medians, and third quartiles. The whiskers represent a 1.5 * inter-quartile range. *P < 0.05; **P ≤ 0.01; and NS, not statistically significant.

From the root samples, we obtained 1,632,424 reads that could be clustered into 200 non-singleton OTUs at 97% sequence similarity, and from the rhizosphere samples we obtained 1,648,165 reads that could be clustered into 736 non-singleton OTUs. Rarefaction analysis suggested that species richness approached an asymptote in roots, but not in the rhizosphere (Supplementary Figure 1). Good's coverage was over 99%, indicating that the obtained sequences represented the majority of the fungal taxa present in the root and rhizosphere samples. Both OTU richness and OTU diversity were significantly higher in the rhizosphere compared to the roots (Figure 3). Significant differences of species richness were found across cultivars both in the roots (*P* = 0.045) and in the rhizosphere (*P* = 0.002). In the roots, species richness significantly differed between plants having no symptoms and plant with severe symptoms in the susceptible cultivar (*P* = 0.016) and in the moderately resistant cultivar (*P* = 0.008), whereas no significant differences could be found in the resistant cultivar. In the rhizosphere soil, cultivars had a significant effect on species richness, but surprisingly, symptom severity did not significantly affect OTU richness. Only species richness in the roots responded significantly to the interactions of both cultivar and symptom severity (*P* = 0.004). The fungal richness was highest in the rhizosphere of the resistant cultivar. No significant differences of microbial diversity were found between plants with or without symptoms, or across cultivars.

### The Structure of Fungal Communities in Roots Is Affected by Symptom Severity

**Figure 4:**
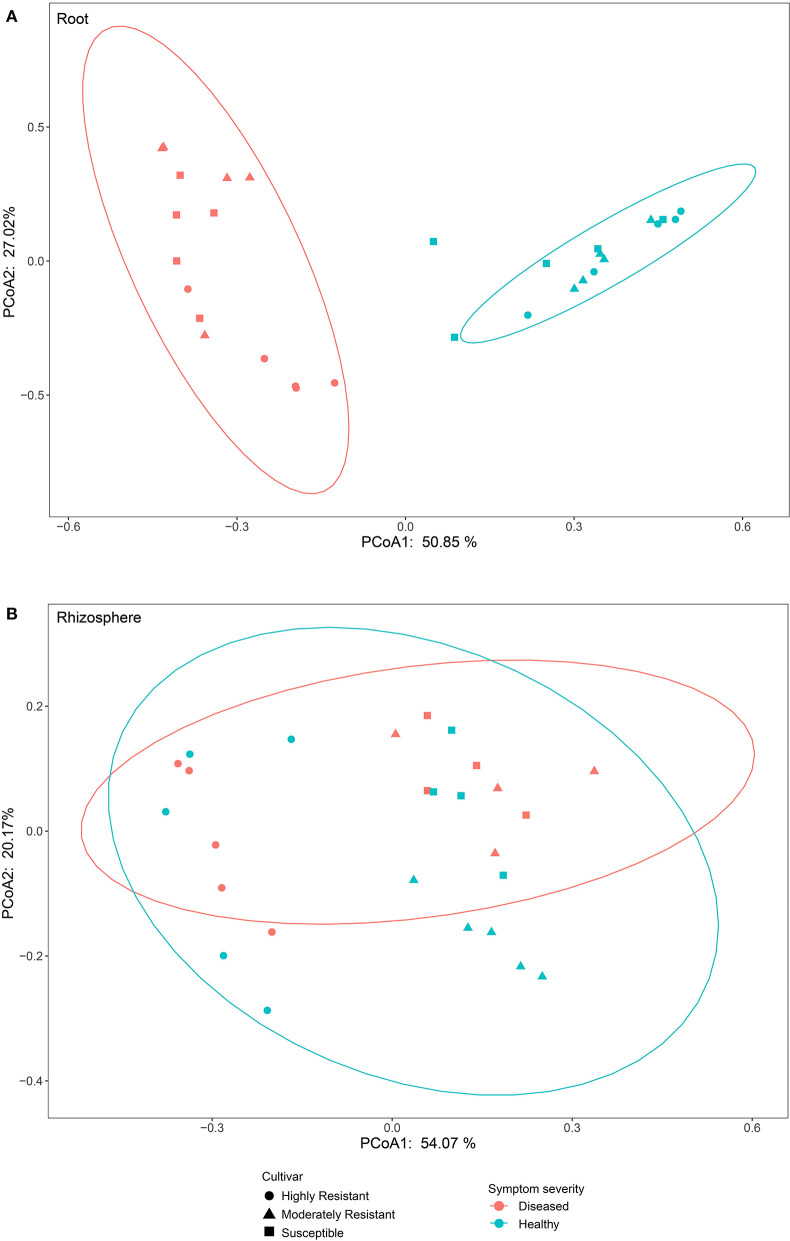
Bray-Curtis matrices visualized using principle coordinates analysis (PCoA) showing distribution of samples according to symptom severity (Diseased and Healthy) and host genotype (Highly resistant, Moderately resistant, and Susceptible cultivars) in roots and rhizosphere soil.

To compare fungal communities among cultivars in roots and the rhizosphere, and to identify the main factors driving community composition, a Bray-Curtis dissimilarity matrix was calculated based on normalized read abundances at OTU level. Fungal community structures among samples were displayed using PCoA (Figure 4). In the roots, distinct clusters were apparent with regard to the symptom severity whereas no clustering could be observed according to cultivar. Rhizosphere fungal communities did not cluster according to symptom severity. However, a clear separation along PC1 was observed between the highly resistant cultivar and the other two cultivars, accounting for 54.1% of the variation. The clustering of fungal communities in the PCoA analyses were statistically supported by ANOSIM and PERMANOVA (Table 1). Overall, both ANOSIM and PERMANOVA revealed that the most significant differences in root fungal communities were between the symptom severity of samples (Global *R* = 0.749 for ANOSIM, *P* ≤ 0.001; *R*^2^ = 0.459 for PERMANOVA, *P* ≤ 0.001). Cultivar only showed significant differences in root samples from plants with symptoms (Global *R* = 0.354 for ANOSIM, *P* ≤ 0.01; *R*^2^ = 0.415 for PERMANOVA, *P* < 0.01). The most striking difference in rhizosphere fungal communities were across cultivars (Global *R* = 0.639 for ANOSIM, *P* ≤ 0.001; *R*^2^ = 0.527 for PERMANOVA, *P* ≤ 0.001). Symptom severity only had significant effects in the moderately resistant cultivar (Global *R* = 0.616 for ANOSIM, *P* < 0.05; *R*^2^ = 0.466 for PERMANOVA, *P* < 0.01). In addition, fungal communities in the roots and rhizosphere responded significantly to the interactions of both cultivar and symptom severity (Table 1).

**Table 1:** Pairwise comparisons of fungal communities based on ANOSIM and PERMANOVA of Bray-Curtis distance for the different factors.

**Factors**	**ANOSIM**		**PERMANOVA**	
	***R*-value**	***P*-value**	***R*^**2**^-value**	***P*-value**
**Root**			
Diseased/Healthy	0.749	0.001^***^	0.459	0.001^***^
Cultivar	0.040	0.156	0.096	0.214
Diseased of all cultivars	0.354	0.01^**^	0.415	0.009^**^
Healthy of all cultivars	0.100	0.143	0.189	0.204
HC vs. MC	0.105	0.081	0.097	0.128
HC vs. SC	0.083	0.119	0.101	0.117
MC vs. SC	−0.055	0.803	0.024	0.255
Cultivar*Symptom severity	/	/	0.097	0.011^*^
**Rhizosphere**			
Diseased/Healthy	0.029	0.225	0.052	0.177
Cultivar	0.639	0.001^***^	0.527	0.001^***^
Symptom severity of HC	0.036	0.293	0.104	0.362
Symptom severity of MC	0.616	0.012^*^	0.466	0.007^**^
Symptom severity of SC	0.174	0.054	0.206	0.07
HC vs. MC	0.890	0.001^***^	0.542	0.001^***^
HC vs. SC	0.906	0.001^***^	0.532	0.001^***^
MC vs. SC	0.156	0.024^*^	0.128	0.032^*^
Cultivar^*^Symptom severity	/	/	0.072	0.024^*^

*ANOSIM, analysis of similarity; PERMANOVA, permutational multivariate analysis of variance. Significance levels*:

* *P < 0.05*;

** P ≤ 0.01; and

*** *P ≤ 0.001. HC, highly resistant cultivar; MC, moderately resistant cultivar; SC, susceptible cultivar*.

### Members of the Mycobiome Are Affected by Symptom Severity and Host Genotype

**Figure 5:**
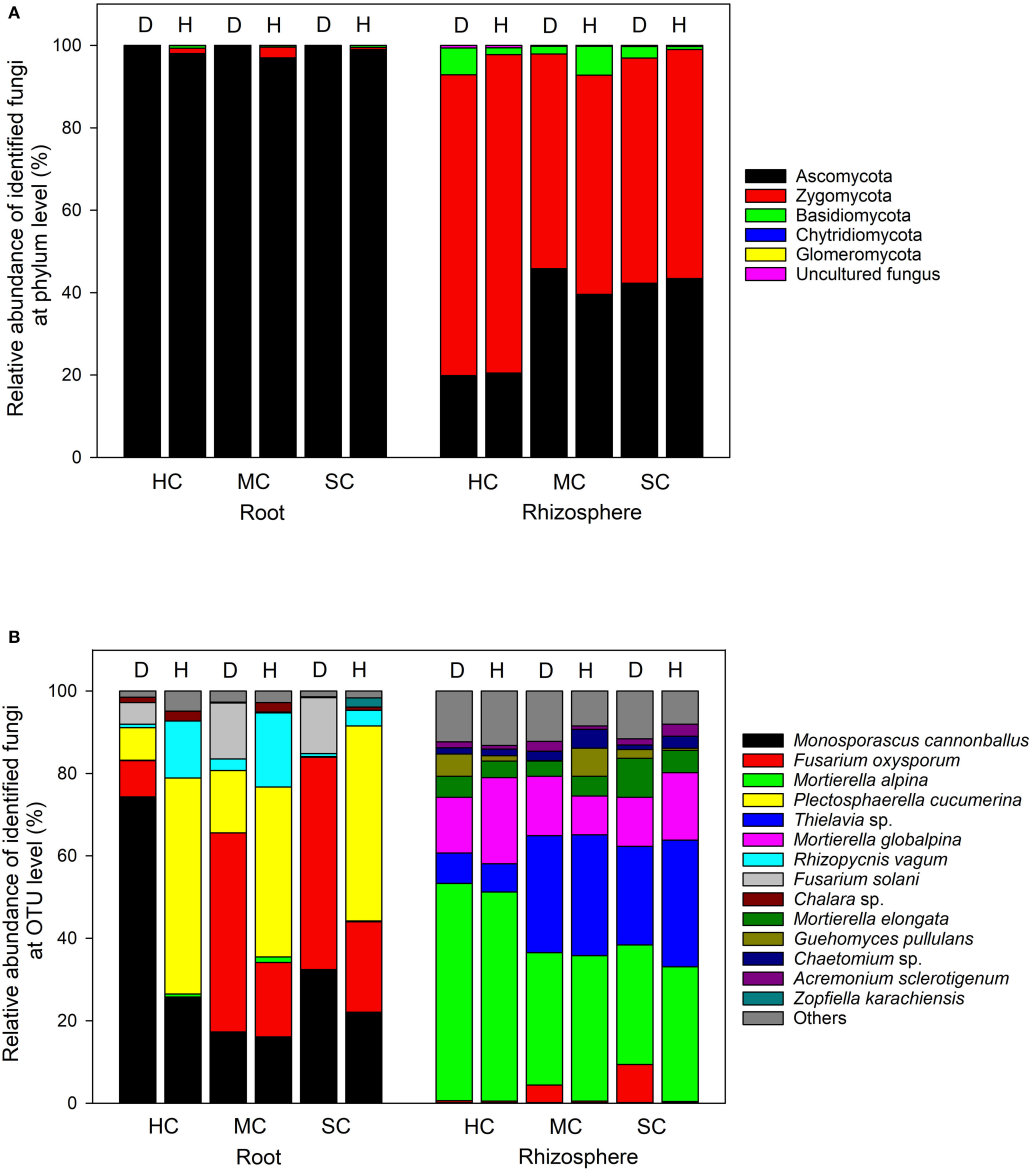
Proportional distribution of different fungal phyla and OTUs in roots and rhizosphere with different symptom severities (D, diseased; H, healthy) and different host genotypes (HC, highly resistant cultivar; MC, moderately resistant cultivar; SC, susceptible cultivar).

In the roots, Ascomycota was the dominant phylum, accounting for more than 96% of the total number of reads, whereas Ascomycota and Zygomycota were the predominant phyla in the rhizosphere soil, with a small quantity of Basidiomycota (Figure 5A). The most abundant taxa accounting for more than 95% (roots) and 86% (rhizosphere) are shown in Figure 5B. *Fusarium oxysporum* reads in the roots, probably identical to FON, was found in different amounts across the three cultivars (*P* = 0.008) and in plant with different symptom levels (*P* = 0.008). *F. oxysporum* reads were more abundant in samples showing symptoms in all three cultivars (susceptible, 48.2%; moderately resistant, 40.9%, and highly resistant, 8.5%). Furthermore, *F. oxysporum* reads were also discovered in the rhizosphere with the highest abundance in plants with symptoms from the two susceptible or moderately resistant cultivars (*P* = 0.009). The overall most abundant OTU in the roots was assigned to *Monosporascus cannonballus*, which varied remarkably across cultivars (*P* = 0.038) and between healthy and diseased plants (*P* = 0.039). It was mostly abundant in the diseased plants (74.3%) compared to the symptom-free plants (25.8%) in the highly resistant cultivar. *Rhizopycnis vagum* found in the roots also had significantly higher relative read abundances in symptom-free samples (*P* = 0.016). In the rhizosphere, *Mortierella alpina* was identified as the most dominant species, and its abundance differed significantly between cultivars (*P* = 0.008) as it was present in higher quantities in the highly resistant cultivars in comparison to the other cultivars. *Thielavia* sp. also had different abundances across cultivars (*P* = 0.037) in the rhizosphere, with lower amount in the highly resistant cultivar in comparison to the other cultivars.

Indicator species analysis identified a range of fungi that responded significantly to the symptom severity of plants and cultivar (Table 2). In the roots, several *Fusarium* spp. were identified as indicators in samples from plants with severe symptoms, whereas 11 OTUs, including several species of *Mortierella*, were associated with samples from plants with no symptoms.

**Table 2:** List of indicator species that were significantly different calculated using the Dufrêne and Legendre indicator species analysis conducted by the indicspecies package in R.

**Symptom severity/Cultivar**	**BLAST ID**	**Indicator value**	***P*-value**
**Root**			
Diseased	*Fusarium oxysporum*	0.985	0.001^***^
	*Fusarium solani*	0.961	0.005^**^
	*Fusarium* sp.	0.930	0.001^***^
	*Fusarium oxysporum*	0.894	0.001^***^
	*Fusarium oxysporum*	0.891	0.002^**^
Healthy	*Mortierella alpina*	0.992	0.001^***^
	*Plectosphaerella cucumerina*	0.992	0.001^***^
	*Cladosporium cladosporioides*	0.990	0.001^***^
	*Candida tropicalis*	0.963	0.001^***^
	*Mortierella alpina*	0.963	0.001^***^
	*Sordariomycetes* sp.	0.959	0.001^***^
	*Mortierella alpina*	0.959	0.001^***^
	*Mortierella* sp.	0.957	0.001^***^
	*Malassezia restricta*	0.949	0.001^***^
	*Penicillium oxalicum*	0.917	0.001^***^
	*Mortierella wolfii*	0.863	0.003^**^
**Rhizosphere**			
Diseased	*Fusarium solani*	0.840	0.042^*^
Healthy	*Oidiodendron periconioides*	0.889	0.001^***^
Highly resistant	*Mortierella hyalina*	0.815	0.018^*^
Moderately resistant	*Blastobotrys mokoenaii*	0.930	0.001^***^
	Uncultured fungus	0.894	0.001^***^
	*Botrytis cinerea*	0.892	0.001^***^
	*Penicillium chrysogenum*	0.887	0.001^***^
	*Trichosporon oleaginosus*	0.862	0.001^***^
	*Cryptococcus podzolicus*	0.810	0.002^**^

*Significance levels*:

* *P < 0.05*;

** P ≤ 0.01; and

*** *P ≤ 0.001*.

### Resistant Cultivars Show Complex Fungal Networks in Roots From Plants With Symptoms

**Figure 6:**
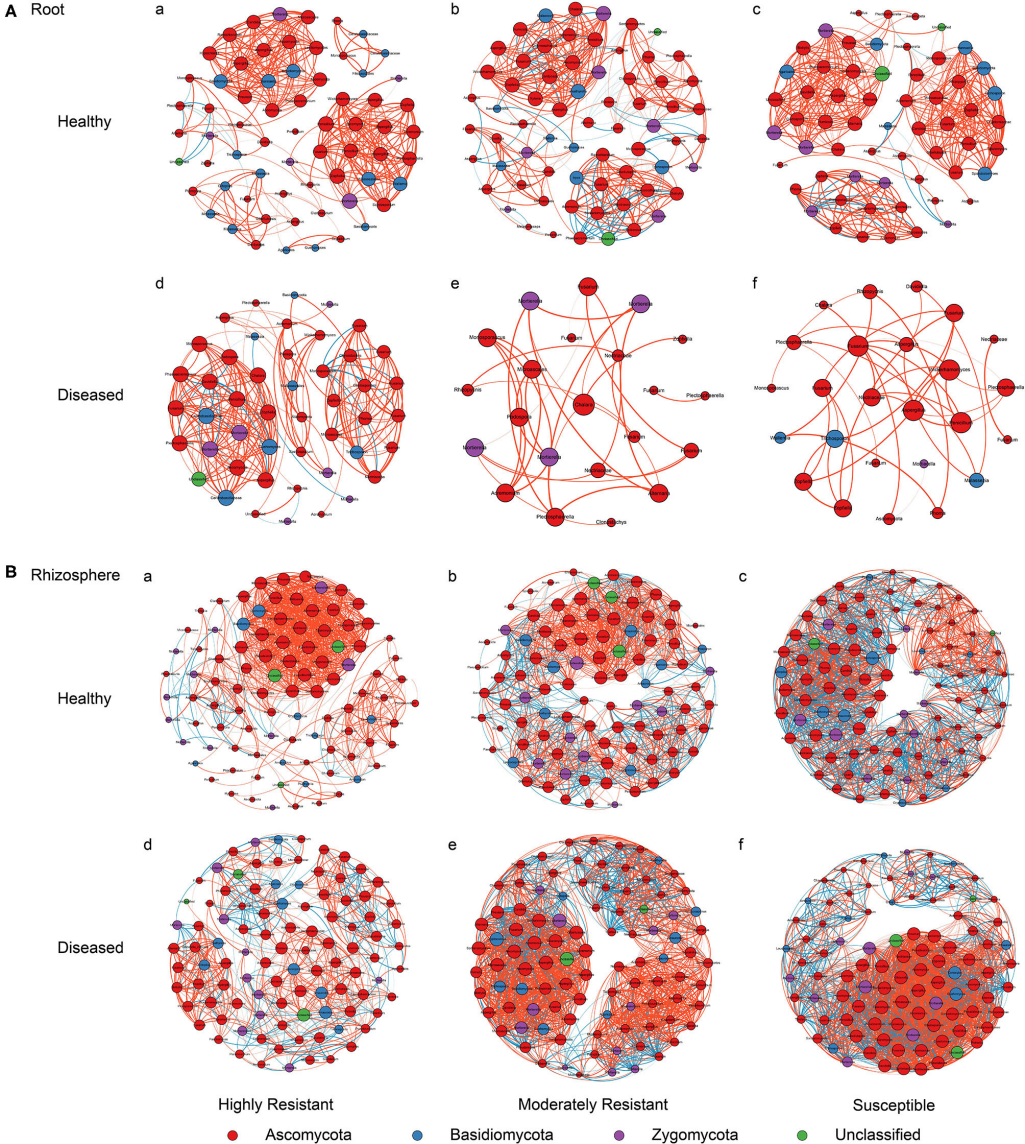
Network co-occurrence analysis of fungal communities in roots and rhizosphere soil. A connection represents a Pearson correlation with a magnitude > 0.7 (red edges: positive correlation) or < − 0.7 (blue edges: negative correlation) which is statistically significant (*P* < 0.05). Each node represents taxa at OTU level. The size of each node is proportional to the number of connections, which is based on ranking of degree with min size = 20 and max size = 50.

A co-occurrence network analysis was performed to explore the complexity of OTU connections within the roots and rhizosphere fungal communities of the different cultivars (Figure 6 and Table 3). Pearson correlations between fungal taxa at OTU level and the topological properties of the obtained networks were calculated to identify differences between samples. Generally, the root microbiomes showed a less complex structure compared to the rhizosphere networks, and they were highly affected by the symptom severity and resistance levels of the host. In the roots, samples from plants with no symptoms showed a more complex structure compared to diseased samples across all cultivars. Remarkably, the roots from the highly resistant plants with symptoms showed a higher complexity of the fungal network compared to the susceptible cultivars. Surprisingly, the rhizosphere of the highly resistant cultivar showed a lower level of complexity and modular structure compared to the susceptible and moderately resistant cultivars. In addition, there were more edges in the network in roots of plants with severe symptoms than in the symptom-free fungal feature network of susceptible and moderately resistant cultivars (Table 3). Overall, the number of positive correlations was much higher than that of the negative correlations in the root and rhizosphere networks. Interestingly, negative correlations were much more abundant in the samples from the highly resistant plants showing symptoms, both in the roots and in the rhizosphere. Fungi with more betweenness centrality (measuring of centrality based on shortest paths) were identified derived from the network properties (Poudel et al., 2016). The betweenness centrality in network analysis indicates the most important nodes as the key taxa inside a connected community (Borgatti, 2005). In the root networks, the key taxa belonging to the genera *Monosporascus* (highly resistant) and *Fusarium* (both moderately resistant and susceptible) were identified in severe symptom samples, respectively, whereas *Penicillium* (highly resistant), *Mortierella* (moderately resistant), and *Trichosporon* (susceptible) were the top nodes in symptom-free samples. In the rhizosphere networks, *Thielavia* (highly resistant), *Acremonium* (moderately resistant), and *Fusarium* (susceptible) were found as key taxa in diseased plants of the different cultivar, respectively, while *Mortierella* (highly resistant), *Pseudaleuria* (moderately resistant), and *Penicillium* (susceptible) were associated with healthy plants.

**Table 3:** Correlations and topological properties of watermelon root and rhizosphere microbiome networks.

**Network properties**	**Highly resistant**		**Moderately resistant**		**Susceptible**	
	**D**	**H**	**D**	**H**	**D**	**H**
**Root**						
Number of nodes^a^	27	66	22	67	25	60
Number of edges^b^	249	316	33	371	37	371
Positive edges^c^	219	309	33	290	37	352
Negative edges^d^	30	7	0	81	0	19
Modularity^e^	0.582	0.678	0.728	0.686	0.741	0.659
Number of communities^f^	4	7	5	5	6	4
Network diameter^g^	8	3	3	9	3	7
Connectivity^h^	9.222	4.788	1.5	5.537	1.48	6.183
Average path length^i^	2.552	1.205	1.273	3.52	1.2	2.462
Average degree^j^	10.596	9.576	3	11.075	2.96	12.367
Average clustering coefficient^k^	0.899	0.889	0.918	0.797	0.821	0.911
**Rhizosphere**						
Number of nodes	99	96	97	97	97	97
Number of edges	672	796	1507	1105	1951	1491
Positive edges	467	761	1139	762	1376	792
Negative edges	205	35	368	343	575	699
Modularity	0.633	0.376	0.533	0.484	0.247	0.470
Number of communities	5	8	3	5	3	3
Network diameter	6	8	4	5	4	4
Connectivity	6.788	8.292	15.536	11.392	20.113	15.371
Average path length	2.902	3.088	2.283	2.386	1.964	2.152
Average degree	13.576	16.583	31.072	22.784	40.227	30.742
Average clustering coefficient	0.663	0.793	0.829	0.714	0.807	0.759

*Different watermelon symptom severity: D, diseased; H, healthy*.

a *OTUs with significant (P < 0.05) and strong (Pearson > 0.7 or < − 0.7) correlation*.

b *Number of connections/correlations obtained by Pearson analysis*.

c *Pearson positive correlation > 0.7*.

d *Pearson negative correlation < − 0.7*.

e *A measure of the structure of network, specifically, the strength of division of a network into modules (Mendes et al., 2018)*.

f *A cluster of nodes densely connected internally*.

g *The longest graph distance between any two nodes in the network*.

h *The beta index measuring the connectivity*.

i *Average graph-distance between all pairs of nodes*.

j *The average number of connections per node in the network*.

k *Embedding in neighborhood*.

## Discussion

Interactions of root-associated fungal communities and plant disease is poorly investigated, and not much is known about the root-associated fungal communities in watermelon and its effect on Fusarium wilt development. In the present study, we studied host resistance, pathogen infection and fungal community interactions in roots of watermelon. We sampled material at the maturation stage of watermelon where disease incidences to our experience usually have stabilized. We characterized fungal communities associated with three watermelon cultivars showing different levels of resistance against FON in plants showing different levels of symptom severity. The three cultivars were consistently infected with FON, but differed in terms of incidence of symptoms and quantities of FON DNA in plant tissues. The quantification of FON by qPCR confirmed visual observations of symptoms of watermelon as also observed by Meng et al. (2019). The qPCR primers specifically targeted the pathogenic strains of FON and excluded non-pathogenic strains of *F. oxysporum* (van Dam et al., 2018). Not surprisingly, the abundance of *F. oxysporum* reads identified by Illumina sequencing showed a similar distribution with significantly higher read abundances in the samples from plants with severe symptoms and from the susceptible and moderately resistant cultivars.

Fungal species richness was significantly affected by cultivar, indicating the importance of genotype in shaping the mycobiome (Berg and Smalla, 2009). In addition, the higher estimated richness in the roots of samples from plants with no symptoms from the susceptible and moderately resistant cultivars probably reflected the dominance of pathogens in the fungal communities of diseased samples. In the roots, high levels of infection (in the moderately resistant and highly susceptible cultivars) were the probable cause of a decrease in OTU richness. The higher richness of fungi discovered in the rhizosphere of the highly resistant cultivar may have lead to a stronger competition for resources contributing to the protection against fungal infections (Wei et al., 2015). Microbial diversity is increasingly recognized as a key factor limiting invasion of pathogens (van Elsas et al., 2012; Mallon et al., 2015).

Fungal communities in roots exhibited a clear clustering according to symptom severity, indicating that pathogen infection is the major factor shaping these communities. This is consistent with previous findings demonstrating the influence of root diseases on fungal communities in the root and rhizosphere of pea (Xu et al., 2012a). Plants are capable of recruiting their rhizosphere microbiome, as evidenced by the fact that specific microbial communities exist in plant species also when grown in identical soil (Berendsen et al., 2012; Wu et al., 2015). Similarly, we observed distinct fungal communities in the rhizosphere of the different cultivars. This is also consistent with previous studies on *F. oxysporum* reporting that rhizosphere microbial community structures of resistant watermelon, common bean, and cucumber cultivars were different from communities in susceptible cultivars (Yao and Wu, 2010; An et al., 2011; Mendes et al., 2018). In contrast, we found that in the roots, host genotype was less important in shaping the fungal communities. One possible reason could be that in roots, pathogens are more important drivers of fungal communities and thus mask potential cultivar effects.

The presence of individual microbial taxa varied strongly in the roots from cultivars with different resistance levels and also between plants with and without symptoms. Several *Fusarium* species were identified as indicators in the diseased roots and also in the rhizosphere samples from diseased plants. These *Fusarium* species have been found in complexes with other fungal pathogens such as *Acremonium cucurbitacearum, Monosporascus cannonballus*, and *Rhizopycnis vagum* causing melon collapse (Martyn and Miller, 1996; Aegerter et al., 2000; Chilosi et al., 2008). This is not unusual as frequently more than one fungal pathogen occur as pathogen complexes associated with the symptoms of the disease (Fitt et al., 2006; Xu et al., 2012b; Lamichhane and Venturi, 2015). On the basis of the high presence of *M. cannonballus* in the diseased roots of the highly resistant cultivar, it may be speculated that the resistance to FON may leave the cultivar more susceptible to *M. cannonballus*, although no visible symptoms of *Monosporascus* infection were observed in this study. *Monosporascus* sp. are adapted to hot, arid and semiarid climates, similar to the climatic conditions in greenhouses (Pivonia et al., 2002). *Rhizopycnis vagum* was more prevalent in healthy than diseased roots. The fungus has been found to have antifungal activities (Lai et al., 2016) and an inhibitory effect toward *F. oxysporum* (Ginting et al., 2013). Nonetheless, *R. vagum* has also been reported associated with watermelon vine decline (Westphal et al., 2011). Several indicator species for symptom-less plants were identified in the roots. *Mortierella* sp., *Penicillium* sp., and *Cladosporium* sp. are known as fungal antagonists possessing a general disease suppressiveness (Van Bruggen and Semenov, 2000; Li et al., 2014; Chaibub et al., 2016).

Co-occurrence networks revealed distinct patterns within the fungal communities of the roots and the rhizosphere that were relatively simple in roots compared to the rhizosphere, as also observed previously (Hartman et al., 2018). Roots from plants with severe symptoms showed highly reduced fungal networks compared to the networks of symptom-less plants. In accordance with this, a long-term increase in disease pressure caused by continuous monoculture of watermelon resulted in a poorly organized microbial community structure (Wang et al., 2019). Moreover, the introduction of a potential pathogen to a community can have important consequences for the network structure, and pathogen invaders can displace key taxa and collapse the network structure (Albrecht et al., 2014). Fewer connections were detected in the network from symptom-free plants than from plants with severe symptoms in the rhizosphere of susceptible and moderately resistant cultivars, as also reported previously (Yuan et al., 2020). The observed higher number of positive correlations compared to negative correlations indicate mutualism between species in complex microbial community ecosystems (Zhang et al., 2014). The more abundant negative edges both in the roots and in the rhizosphere of the highly resistant cultivar may suggest the presence of predation and competition (Chow et al., 2014). Competition has proved to be a major type of interaction between microorganisms, and plays a key role in suppression of soil-borne plant diseases (Li et al., 2020). Invading fungal pathogens induce stress responses in the rhizosphere microbiome and induce shifts in the community composition and activation of antagonistic traits (Chapelle et al., 2016). Interestingly, the key species identified in the network from plants with severe symptoms were mostly pathogens, whereas the key taxa associated with the fungal network of the symptom-less plants were mostly beneficial fungi, particularly *Mortierella* sp. This fungus is important in vanilla Fusarium wilt disease suppression (Xiong et al., 2017), and was also identified as a dominant plant-benefial fungus in soil consecutively cultured with peanut (Li et al., 2014).

## Conclusions

We demonstrated that host resistance is a major driving force in shaping the rhizosphere microbiome, whereas a significant role of FON infection on root microbiomes was found. Plant disease and host resistance had dramatic effects on fungal networks in the roots and several species that were dominating the communities from the healthy plants were identified. We thus speculate that fungal communities may play an important role in controlling FON infection in the highly resistant cultivar. Our results highlighted the importance of fungal communities in determining the outcome of plant-pathogen interactions and may thus contribute to future plant breeding strategies.

## Data Availability Statement

The datasets generated for this study can be found in online repositories. The names of the repository/repositories and accession number(s) can be found in the article/Supplementary Material.

## Author Contributions

LX and FD designed the experiment. LX, RZ, and SG sampled and performed the laboratory work. LX, MN, RZ, and SG analyzed experimental data. LX, MN, and JL prepared the manuscript. All authors approved the manuscript for submission.

## Conflict of Interest

The authors declare that the research was conducted in the absence of any commercial or financial relationships that could be construed as a potential conflict of interest.

## Supplementary Material

Supplementary Figure 1 Rarefaction curves depicting the effect of total number of sequences sampled on the number of OTUs identified from the roots and rhizosphere of three watermelon cultivars (HT, roots of highly resistant cultivar; MT, roots of moderately resistant cultivar; ST, roots of susceptible cultivar; HR, rhizosphere of highly resistant cultivar; MR, rhizosphere of moderately resistant cultivar; SR, rhizosphere of susceptible cultivar).
