# Supplementary figures and images for: Pathogen Infection and Host-Resistance Interactively Affect Root-Associated Fungal Communities in Watermelon
Source: Front Microbiol. 2020 Dec 17;11:605622. doi: 10.3389/fmicb.2020.605622 (PMC7793699; doi:10.3389/fmicb.2020.605622)

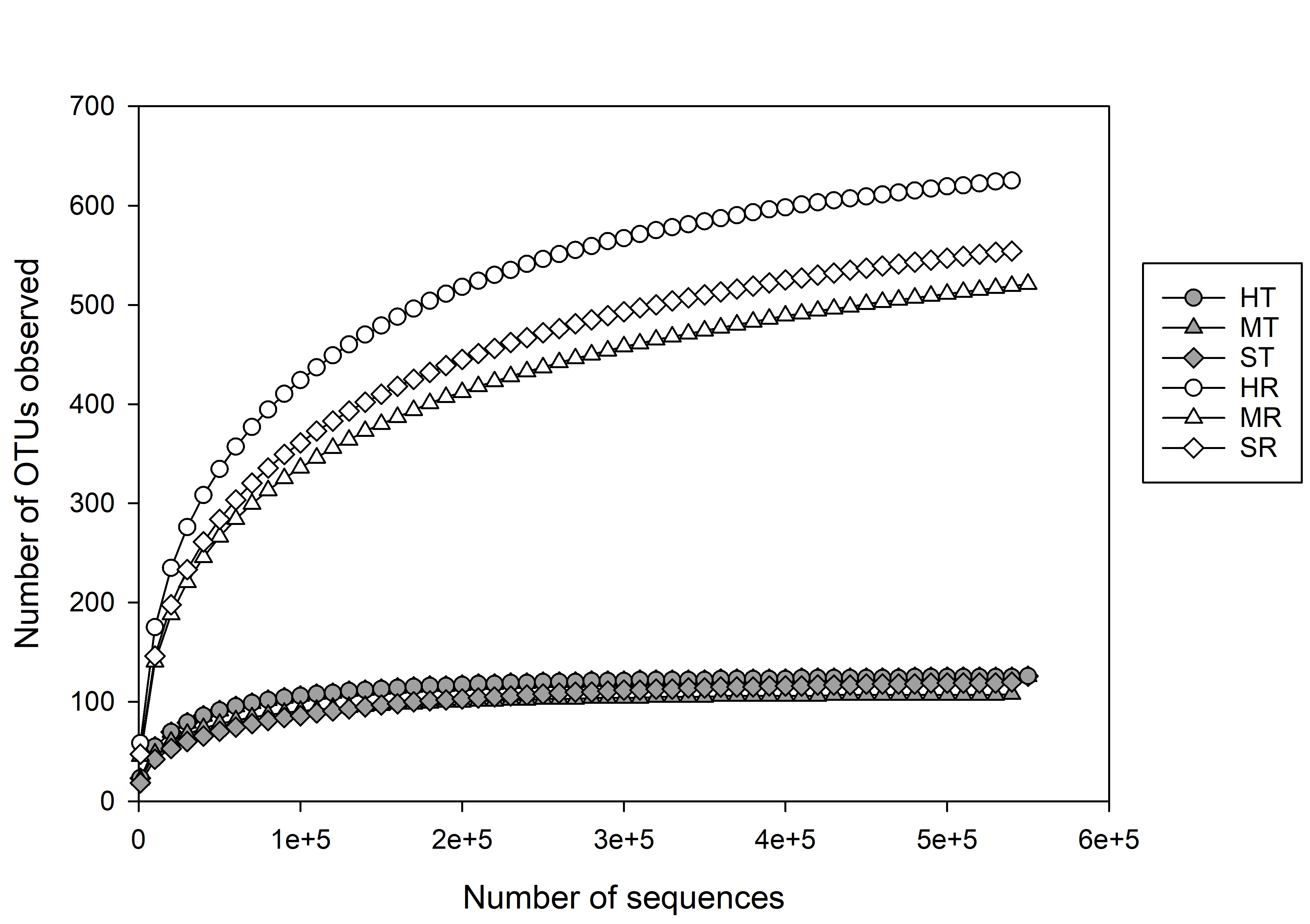

Supplement: Supplementary Figure 1 — Rarefaction curves depicting the effect of total number of sequences sampled on the number of OTUs identified from the roots and rhizosphere of three watermelon cultivars (HT, roots of highly resistant cultivar; MT, roots of moderately resistant cultivar; ST, roots of susceptible cultivar; HR, rhizosphere of highly resistant cultivar; MR, rhizosphere of moderately resistant cultivar; SR, rhizosphere of susceptible cultivar). [file Image_1.JPEG]
